# Confronting the quality paradox: towards new characterisations of ‘quality’ in contemporary healthcare

**DOI:** 10.1186/s12913-015-0851-y

**Published:** 2015-06-19

**Authors:** Deborah Swinglehurst, Nathan Emmerich, Jo Maybin, Sophie Park, Sally Quilligan

**Affiliations:** Centre for Primary Care and Public Health, Queen Mary, University of London, London, UK; School of Politics, International Studies & Philosophy, Queen’s University Belfast, Belfast, UK; Policy Directorate, The King’s Fund, London, UK; Department of Primary Care & Population Health, Institute of Epidemiology & Health, University College London, London, UK; Clinical and Communication Skills Unit, School of Clinical Medicine, University of Cambridge, Cambridge, UK

## Abstract

This editorial introduces the special Biomed Central cross-journal collection *The Many Meanings of ‘Quality’ in Healthcare: Interdisciplinary Perspectives,* setting out the context for the development of the collection, and presenting brief summaries of all the included papers in three broad themes 1) the practices of assuring quality in healthcare 2) giving ‘space to the story’ 3) addressing moral complexity in the clinic, the classroom and the academy. The editorial concludes with reflections on some of the key messages that emerge from the papers which are relevant to policymakers and practitioners who seek to improve the quality of healthcare.

## Editorial

This special Biomed Central cross-journal collection *The Many Meanings of ‘Quality’ in Healthcare: Interdisciplinary Perspectives* presents papers written by authors with a range of disciplinary backgrounds (spanning medicine, social policy, anthropology, accountancy, philosophy, design, to name a few) whose work engages directly with notions of ‘quality’ in healthcare. The inspiration for this collection began with a colloquium of the same title which we organised in June 2013 at Cumberland Lodge, Great Windsor Park, UK [1,2]. This event brought together health professionals, academics, policymakers and patient representatives (predominantly but not exclusively UK-based) to unpack the notion of ‘quality’ in healthcare, offering an opportunity to share insights, and to discuss and debate contemporary programmes of quality implementation, evaluation and improvement. We wanted to identify some of the key processes and attributes of quality in healthcare, and explore the professional values required to support and improve it. We were also keen to discover what might emerge when scholars from a diverse range of disciplines were provided with a critical space in which to wrestle with an important, controversial and politically charged topic. We are delighted that we have been able to capitalise on the ongoing enthusiasm and critical insights of the colloquium presenters and their co-authors in this collection.

The assiduous pursuit of ‘quality’ (usually defined with terms such as safety, effectiveness, and positive patient experience [3,4]) is actively shaping delivery of healthcare both nationally and internationally [5]. The UK has been at the forefront of many such quality initiatives. Examples include: the introduction of the Quality and Outcomes Framework in 2004 (an incentive scheme that rewards general practitioners financially for demonstrating achievement across a range of clinical and organisational indicators); the compulsory revalidation of doctors since 2012 (revalidation of nurses and midwives is planned from 2016), and the mandatory inspection of health and social care services by an independent regulatory body, the Care Quality Commission, which publishes its findings as performance ratings.

Given the prominence of the term ‘quality’ across health policy and practice and its centrality to many international efforts to improve healthcare, it is perhaps surprising to suggest that what we might *mean* when we speak of ‘quality’ remains obscure and worthy of debate [6]. However the case for pausing to reconsider and extend our understandings of ‘quality’ is strong, especially since it is increasingly recognised that the pursuit and measurement of quality in healthcare often fails to deliver anticipated benefits and may have perverse consequences, undermining rather than strengthening the characteristics it seeks to assure and requiring excessive data gathering by healthcare staff [7–11]. This ‘quality paradox’ resonates through the papers published in this special issue and is, in essence, the ‘wicked’ problem that we seek to illuminate and explore [12]. Although the point of departure is healthcare activity in the UK, the papers speak to the issue of ‘quality’ in ways which are likely to be of relevance to policymakers and practitioners internationally.

The collection brings together both empirical research papers and articles that primarily offer theoretical engagement and analysis. Whilst some authors grapple with fundamental conceptual concerns, others explore the practical ways in which ‘quality’ may (or may not) become enacted in the real world of healthcare. We believe that the value of the special issue lies not only in the rich variety of perspectives it presents, but in appreciating the connections, synergies and lines of argument as they play out *across* the collection. Therefore, we strongly encourage readers to put aside disciplinary allegiances and consider straying beyond the articles that seem most immediately appealing. Whilst there are doubtless many alternative ways of categorising the papers by theme, and any such categorisation overlooks areas of overlap and linkage, we will summarise the papers in three broad groupings: the practices of assuring quality in healthcare; giving ‘space to the story’; and addressing moral complexity in the clinic, the classroom and the academy.

### 1. The practices of assuring quality in healthcare

One important theme to emerge from the papers is how the principles and systems which are intended to assure and improve the quality of healthcare are realised in *practice*. These papers emphasise the need to attend to the everyday activities of patients, carers, clinicians and administrators in producing and accounting for high quality care in order to fully grasp their meanings and consequences.

Simon Cohn’s paper focuses on the concept of ‘trust’ in healthcare, which has received renewed attention as a possible counter-balance to the dehumanising effects of audit and market based processes for quality assurance. Challenging the common conception of trust as a psychological state resulting from deliberative evaluations of an individual’s behaviour, Cohn draws on ethnographic data from a UK diabetes clinic to show how in this context, trust constitutes a ‘sense of stability and predictability’ for patients which emerges from a particular set of relationships between people (extending beyond the doctor-patient dyad to include family members), physical objects (such as patient notes) and the wider material environment [13]. He warns that singular, generalizable concepts or measures of trust will necessarily overlook the context-specific, dynamic and contingent qualities of these relationships which give meaning to the trust concept.

Dane Pflueger’s paper makes the case for paying equivalent attention to the ways in which data are produced and used in dominant approaches to accounting for quality in healthcare [14]. Pflueger sets out how tools such as the patient experience survey programmes and the UK Quality and Outcomes Framework are based on a concern to ‘capture information, as a camera might’. In this context quality improvement efforts become focused on perfecting the technical process of data collection (‘developing sharper and better lenses’ by adjusting data for case mix, for example), but managers and others fail to attend to the ways in which such data get produced in practice. Drawing on insights from the ‘social studies of accounting’ literature, Pflueger describes how accounting ‘does not just find things out but makes them up’, as particular tools impose certain sets of ‘preoccupations and objectives’ in order to make things knowable. Pflueger does not suggest abandoning such approaches but rather calls for a more sociologically-informed approach to the use of accounting, for example, cultivating a ‘skeptical calculative culture’ in which data about care quality are used as an invitation to discussion rather than a prescription for action.

Swinglehurst and Greenhalgh’s paper on the production of care records in general practice provides just such an example of the benefits of attending to the practices by which data for audit are produced [15]. Drawing on an ethnography of the work of administrators in two GP practices, the authors demonstrate how in these cases coding patients’ medical records was not principally a matter of ‘capturing’ specific diagnoses in code form, but instead comprised nuanced acts of interpretation, judgement and relationship-building in order to produce a record which met the multiple and potentially conflicting needs of doctors, patients and managers.

Greenhalgh and colleagues’ paper on quality in telehealth and telecare provides an example of rooting quality standards in the lived experience of service users and providers [16]. Using the principles of experience-based co-design, the authors collected ethnographic data on service users’ and carers’ experiences of illness and assistive technologies, and drew on interviews with healthcare providers and technology designers. The gathered insights formed the basis of a collaborative workshop in which the participants collectively developed a set of ‘design principles’ for telehealth and telecare products and services. The project found that ‘assistive technologies will always need skilled human work, inter-sectoral negotiation and *social* infrastructure to ensure that they “work”’, and developed a framework to support the implementation of these principles in the design and delivery of services.

The papers within this theme make a number of important contributions to contemporary policy debates about how to improve quality in healthcare. They illuminate how and why efforts to develop processes and products intended to *ensure* against quality problems necessarily fail in their ambitions to certainty, and can in fact be insidious. They emphasise how all systems, processes and technologies are in practice socially negotiated, and warn that a failure to attend to this fact can result in ineffective policies and unintended consequences. The papers also illustrate that while it is necessary to recognise the limitations of dominant rational systems of audit and management, it is important to move beyond pitching the ‘rational’ against the ‘real’, or ‘systems’ against ‘human’ approaches, and to attend instead to how people can and do overcome the dysfunctional and counterproductive effects of dominant approaches to assuring the quality of healthcare in practice.

### 2. Giving ‘space to the story’

The papers in this theme focus on the gap between what is easily measurable and what is *meaningful* in relation to the quality of healthcare, and critique the tendency for dominant approaches to quality evaluation to privilege the former.

Kelly et al. provide a practical illustration of using narrative and interpretation to bridge the world of biomedicine and the patient’s life-world [17]. They report on a project which used sewing workshops with local Bangladeshi women as a site for developing conversations about key health issues such as the importance of vitamin D. Through art and story-sharing the women found a way to explore issues that might not otherwise have been discussed or heard within the constraints of the consulting room. While the project outcomes may be difficult to measure, participants gained confidence through sharing stories and experiences, and group discussion gave precedence to the patients’ narratives, while still enabling clinicians to share their own expertise. The authors conceptualise the quality of these encounters in terms of ‘ensuring a space for the story’ for both patients and professionals.

The potential value of offering staff and patients the opportunity to share stories and experiences is also explored in Thomson’s paper [18]. Thomson describes an innovative outpatient project that used design-led, participatory methods based on the metaphor of the patient’s journey to help patients with multiple sclerosis and health care staff to develop a collective vision of how care could be improved in the outpatient department in the future.

Renedo and Marston highlight a particular challenge that may arise from efforts to involve patients in quality improvement initiatives. Drawing on ethnographic observation and analysis of interviews with patients engaged in this work, the authors advise practitioners to be aware that neoliberal ideals and rationalities dominant in policy and service delivery contexts may shape patients’ own accounts of what quality improvement means (or should mean) in practice [19]. They report that although patients’ accounts initially focused on quality improvement as dependent on collective action, they quickly shifted towards an emphasis on the responsibilities of the individual patient to improve their own health. This finding resonates with Pflueger’s suggestion that how quality is accounted for and operationalised in dominant health policy discourse actively shapes practices on the ground in ways that can be constraining as well as enabling [14].

Papers by Tomlinson, and Farr and Cressey, also address the need to ensure a space for the story, this time with a particular focus on professionals’ narratives [20,21]. Authors of both papers argue that this space should be one in which the tacit, intangible and relational dimensions of care can be voiced, and heard, as important aspects of quality in practice. Through in-depth qualitative interviews with health professionals and managers, Farr and Cressey conclude that staff values and personal and professional standards are core to understanding how quality is co-produced in service interactions, challenging us to consider that what emerges in practice may not always align with standard performance metrics and may, at times (and for good reason) be at odds with accepted ‘best practice’ [21]. Tomlinson suggests that one way to access these values and standards is through narrative clinical supervision, which can foster a culture that is supportive, educational, self-critical, outward-looking, patient-focussed, and centred on patient safety and quality care [20].

Millar et al. present findings from an interview study in which they analysed the narratives of national NHS policy actors on the role of hospital board oversight of quality and safety [22]. The authors weave these narratives around two key themes developed from the academic literature on assuring quality: ‘trust’ and ‘intelligence’. In particular the authors argue for the importance of triangulating different forms of intelligence to inform quality and safety activities, bringing together performance data (such as hospital acquired infection rates) with ‘softer’ forms of intelligence gained through feedback and interactions with staff, patients and the public. They call for a form of trust that goes beyond usual metrics and proactively engages the qualitative dimensions of hospital life, “characterised by styles of leadership and behaviours that are attentive to the needs and concerns of both staff and patients”.

The papers in this section challenge normative understandings of quality, that is the ‘taken-for-granted’ ways in which ‘quality’ tends to be conceptualised and ‘talked about’. For these authors, a full appreciation of ‘quality’ means paying close attention to the unmeasurable, relational and (relatively) ‘hidden’ dimensions of care by seeking to connect with the values, culture and life-worlds of patients and staff. ‘Stories’ are one important means of achieving this.

### 3. Addressing moral complexity in the clinic, the classroom and the academy

The papers in this final theme all focus on the ‘ethical’ in order to tackle notions of quality from a distinctly normative perspective. The first paper by Emmerich et al. suggests that we should reflect on the ethical consequences of quality of care discourses and the instruments of governance that have arisen in their wake [23]. Concern for the quality of care has become translated into particular bureaucratic processes that seek its assurance through audit. Emmerich et al. argue that bodies like the Care Quality Commission (CQC) are therefore predisposed to privilege what is auditable over what is not. The reflexive nature of social reality means that institutions that are subject to such audits respond by prioritising the phenomena that are measured by ‘quality of care’ instruments. This can have adverse consequences for quality of care as what is not, or cannot be, subject to audit and evaluation may become neglected, deprioritised and otherwise compromised; ‘quality of care’ audits can be seen as ‘symbolically violent’. In a message that resonates with that of Pflueger [14], the authors do not recommend abandoning the audit approach entirely. Rather, they suggest that there is an ethical need to acknowledge the limitations of audit and work to augment and modulate the ways in which quality of care is evaluated and subsequently managed.

Stevenson et al. engage with the ethical review of research from the perspective of its quality [24]. In recent times there has been a growing concern about the way in which processes and procedures of ethical governance impact on research, especially qualitative social science research [25]. The authors begin with what is, by now, a reasonably familiar point: qualitative research tends to be poorly received by Research Ethics Committees (RECs) and IRBs (Institutional Research Boards). Not only has this observation failed to drive any real change to the approach taken to ethical review, but the authors present evidence of a more worrying concern - that the quality of some types of research may be compromised *because of* the failure to revise the approach taken to ethics review. Drawing on their own experience with the UK’s National Research Ethics Service (NRES), Stevenson et al. argue that the current ‘pre-dictive’ approach to review fails to recognise one of the key virtues of a qualitative approach - its fluid or iterative nature, whereby the research process is responsive to insights generated as the research proceeds. As such, the review process impacts negatively on the quality of qualitative research. The authors do not call for an end to ethical review but suggest an alternative approach which they call ‘iterative ethics’. This would, they argue, allow researchers to engage with review committees without compromising the integrity of qualitative research or the ethical relationship between researchers and research participants. In short, Stevenson et al. identify an urgent need to reflect critically on the ethics of ethical review.

Wintrup also draws our attention to an important problem which is not usually addressed in ethics education for clinicians. She is critical of the tendency of training courses to focus on solving problems, with insufficient attention paid to the complex, ‘contested arenas’ or broader ‘imperfect’ socio-political contexts in which ethical problems in healthcare occur [26]. Wintrup recommends that we reject the tendency to simplify cases, instead embracing the ‘case’ as a complex, socially situated and constructed phenomena, influenced by broader structural conditions and macro political arrangements of power and influence. She urges educators to embrace these issues and suggests that in doing so healthcare professionals will become more inclined to consider and engage with the ethico-political questions raised by the provision of health care. This, she suggests will contribute to securing a quality healthcare service for the future.

In the final paper within this theme, author Iona Heath opens with the challenging and thought-provoking statement that quality is a contemporary catchword “that seldom has real substance and all too often become a slogan for the exercise of power” [27]. Quality assurance and evaluation, she argues, have become an irresistible machine that bends practice to its will. This machine absorbs the time, attention and resources of frontline practitioners and does so largely in the interests of conformity and standardization, objectifying both patient and doctor and ignoring all that is unique in their human subjectivity. For example, Heath points out that the Quality and Outcomes Framework applied to UK General Practice is designed on the basis of average or ‘standardized’ patients, and constructed on the basis of populations without co-morbidities or other complicating factors, a model which she suggests is, at best, inappropriate. Like several other authors in this collection she is also critical of over-reliance of quality assessment on quantitative data: “numbers have taken the place of words” as our understanding of quality has been restricted by our preference for quantification. She ends on a salutary note: “we waste effort, money and time collecting data and pursuing quality targets so that we have less time to listen and we risk losing sight of the human suffering subject”.

## Moving beyond the quality paradox

In common with other ‘wicked’ problems [12] there is no single simple solution to the ‘quality’ of healthcare conundrum. However, the clear message from this collection is that the assumptions which underpin many, if not most, current approaches to managing the ‘quality’ of healthcare lie open to contest. How quality is often *imagined* in the minds of health policy makers and practitioners is not – it would seem - how it comes to *be.* Several of the papers in this collection offer promising suggestions for more constructive approaches to improving the ‘quality’ of care providing some glimpses of possible alternative practices. They challenge us to be more imaginative in how we ‘measure’ quality and more committed to seeing any such ‘measurement’ as a starting point in the project of characterising care in the context of complex, inherently unpredictable environments populated by unique individuals with unique needs [23,24,27]. Suggestions that arise from this collection include fostering a ‘skeptical calculative culture’ [14], and giving more explicit consideration to what measurement practices such as audit are capable of revealing, concealing and producing [14,23], and triangulating different forms of data, deliberately seeking out connections and disconnections between them [14,22]. We are reminded that quality of care involves arrangements of people, practices and materialities that go beyond the patient-clinician dyad [13,15,16,22], and that standards of care quality must be rooted in the lived experience of patients, their carers, clinicians and managers [16-18,21,22], rather than on impoverished proxy representations. Finally, there is a moral imperative to ensure that professional education embraces the morally complex, messy realities of patients’ lives and socio-political contexts within which patients and practitioners interact [20,26,27].

We would like to express our grateful thanks to the editors of the six participating journals at *BioMed Central* (*BMC Medicine; BMC Health Services Research; BMC Medical Ethics; BMC Family Practice; BMC Medical Education; Philosophy, Ethics and Humanities in Medicine*) for making possible this cross-journal special issue by utilising their Open Access online publishing space. We can think of few other platforms that would bring together such an eclectic group of papers, and hope that this project may sow a seed for similar publishing ventures that connect otherwise dispersed and seemingly ‘unconnected’ facets of wide ranging topics such as ‘quality’ in healthcare.
